# The effect of long-term care insurance on healthcare utilization of middle-aged and older adults: evidence from China health and retirement longitudinal study

**DOI:** 10.1186/s12939-023-02042-x

**Published:** 2023-10-30

**Authors:** Songhao Yang, Dandan Guo, Shengxian Bi, Yingchun Chen

**Affiliations:** 1 Department of Health Management, School of Medicine and Health Management, Tongji Medical College, Huazhong University of Science and Technology, Wuhan, 430030 China; 2 Key Research Institute of Humanities & Social Sciences of Hubei Provincial Department of Education, Research Centre for Rural Health Service, Wuhan, 430030 China

**Keywords:** China, Difference-in-differences propensity score matching estimator method, Health care utilization, Long-term care insurance, Middle-aged and older people

## Abstract

**Background:**

As global ageing continues to increase and many countries face challenges from the growing demand for long-term care. Drawing on the experiences of developed countries, developing countries have explored their own suitable long-term care insurance and have shown strong potential for development and research prospects. However, due to their late start, relevant research is underrepresented in the global research network and still needs to be supplemented. The present study hopes to examine the effect of long-term care insurance on healthcare utilization among the middle-aged and elderly from an empirical perspective, using China as an example.

**Methods:**

Panel data from wave 3 (2015) and wave 4 (2018) of the nationally-representative China health and retirement longitudinal study were selected to obtain a sample of 661 processing participants and 16,065 control participants after matching the policy implementation time in the first pilot cities, and quantitative analysis was conducted using difference-in-differences propensity score matching estimator method to assess the net effect of long-term care insurance on health care utilization among the middle-aged and elderly adults.

**Results:**

In the matched frequency-weighted regression difference-in-differences estimator results, long-term care insurance had a negative effect on the number and costs of annual hospitalizations at the 5% significance level (key variable values of − 0.0568101 and − 1236.309, respectively) and a non-significant effect on outpatient service utilization (*P* > 0.05). Further exploration of the heterogeneous effect of it revealed that implementation had a more significant negative effect on hospitalization utilization for middle-aged and older people in the East and for those with higher levels of education or attended care.

**Conclusion:**

Long-term care insurance has played a role in controlling hospitalization costs but has not yet achieved the expected effect in controlling outpatient costs. The policy effects in terms of regional distribution and education level and care situation have been variable. The treatment plan of long-term care insurance needs to be improved, the supply of resources for long-term care services should be increased, and the promotion of long-term care insurance and health science should be given attention.

## Background

In recent years, the world’s ageing population has grown rapidly. According to the *World Population Prospects* (2020 Revision), the number of people aged 60 and over in 2020 reaches 1.05 billion, representing 13.46% of the world’s total population. This number is estimated to reach 1.41 billion (16.46%) in 2030 and over 2 billion (22%) in 2050. Ageing has become a huge worldwide problem [1, 2]. As the elderly age, their body functions and resistance to environmental stresses diminish, increasing their need for medical and daily care [3], so middle-aged and older people make up the majority of disabled people. The increase in the number of disabled adults leads to a decline in their quality of life and life satisfaction, and puts pressure on their family members and finances, it also leads to increased burden on social health resources [4–6]. Long-term care insurance (LTCI) was created to meet the demand for long-term care brought about by the increase in the number of disabled people, and Japan, Germany, the United States and other countries with serious ageing have started to implement and explore it.

LTCI is a type of health insurance that provides reimbursement for various expenses incurred by insured persons who are unable to engage in daily activities because of age, illness and disability and who need to receive various daily basic care and rehabilitation services from others at home or in a nursing home for a relatively long period [7]. Given that more and more countries began to explore LTCI models to adapt to their respective national conditions, research on LTCI has experienced significant growth in the past 20 years, the focus of scholarly research on LTCI has evolved over time and at different times. On the one hand, the focus of scholars has shifted from the financing and market construction and reform of LTCI to the needs and actual effectiveness of the insured, with the research trend gradually shifting from the supply side to the demand side [8]. On the other hand, many developing countries are also beginning to look into establishing LTCI that suits their national circumstances, and the number of relevant research is increasing. Although these countries started late in exploring LTCI and relevant research is underrepresented in global research networks, they have explored the LTCI suitable for their own countries based on the experience of developed countries, and have shown strong development potential and research prospects in this area [9].

With one-fifth of the world’s elderly, China faces the greater challenge of population ageing, and the increased form of ageing will be met with more serious problems of increased disability and increased social burdens. Therefore, relevant research is necessary and can provide some reference for other developing countries [10]. According to the China Development Report 2020: Ageing Population: China’s Development Trend and Policy Options by the China Development Research Foundation, China’s population aged 65 and above is 190.64 million, accounting for 13.50% of the country’s total population. According to short-term projections, China will have entered an aged society by 2022 (based on a proportion of 14% to 20% of the population aged 65 and above) and will enter a super-aged society by 2033 (based on a proportion of 20% or more of the population aged 65 and above) [11]. ‘Living with illness’ is a common phenomenon among older people, but the current Chinese basic medical insurance covers the average medical needs of residents. It does not specifically consider the characteristics of elderly people regarding healthcare behaviour and medical cost distribution. The design of the medical insurance system rather than health protection has not kept pace with the development trend of ageing and changes in the disease spectrum [12]. Data from the China Health Insurance Research Association sample in 2017 show that only 17.3% of the population aged 60 and above participate health insurance, but the total expenses reimbursed by these people through basic health insurance account for nearly 50%, with inpatient medical expenses accounting for 53.3%. Pressure on the health insurance fund will become more pronounced as the degree of ageing increases. At the same time, China suffers from a relatively small number of elderly care institutions and a shortage of professional caregivers. The disability of middle-aged and elderly people lead to increased demand for medical and health services, resulting in the higher burden on medical resources. Coupled with the high number of one-child families in China, the traditional model of relying on families to provide informal care is unsustainable [13]. Therefore, China must take effective measures immediately to prevent ageing before preparation.

Against this backdrop, China has decided to explore LTCI, which can meet the care needs of disabled middle-aged and elderly people and is compatible with the domestic health insurance system. Given China’s enormous size and regional social and economic differences, applying a one-size-fits-all approach is challenging. Therefore, as with many reforms in other policy areas [14], China has decided to develop LTCI through policy experimentation. Under an experimentation framework, the Chinese central government encourages sub-national governments to undertake pilot projects to solve problems and explore new policy options through trial and error and to gain experience through pilot projects to help the central government find policies that can be applied nationally [15]. China launched a pilot scheme for LTCI in 15 cities and two key provinces in 2016 rather than immediately starting a national policy pilot scheme, the implementation time, population covered and eligibility of the LTCI pilot programme in each pilot city are shown in Table 1. The higher the degree of disability,the lower the ability to take care of oneself, and the higher the demand for long-term care [16], so the people in need of long-term care examined in this research are mainly people with severe incapacity. Some pilot cities have also extended coverage to people with moderate disability as well as those with dementia. However, the tools used to assess the degree of disability were not identical across cities, with some pilot cities choosing the Barthel Scale and others choosing a locally developed composite scale. In 2020, more than 18.93 million disabled people will be over 60 in China, accounting for 7.45% of the elderly population over the age of 60. Among them, more than 7.09 million will be moderately or severely disabled, with expenditures of 16.84 billion yuan for the use of LTCI [17].

**Table 1:** Timing of implementation, coverage and eligibility for the first LTCI pilot areas

Pioneer Cities	Execution Time	Target Population			Eligibility to Benefits			
		Employee	Urban Residents	Rural Residents	Severely Disabled	Moderately Disabled	Mild Disabled	Intellectually Disabled
Chengde	Nov. 2016	√			√			
Changchun	May 2015	√	√		√			
Jilin	Nov. 2016	√	√		√			
Qiqihaer	Oct. 2017	√			√			
Shanghai	Jan. 2017	√	√and ages 60 +	√and ages 60 +	√	√		
Nantong	Jan. 2016	√	√	√	√	√		√
Suzhou	June 2017	√	√	√	√	√		
Ningbo	Dec. 2017	√			√			
Anqing	Jan. 2017	√			√			
Shangrao	Nov. 2016	√			√			√
Jinan	Jan. 2016	√			√	√		
Qingdao	July 2012	√	√	√	√	√		√
Weifang	Jan. 2015	√			√			
Liaocheng	Oct. 2017	√			√			
Binzhou	Jan. 2018	√			√	√		
Jingmen	Nov. 2016	√	√	√	√			
Guangzhou	Aug. 2017	√			√	√		√
Chongqing	Dec. 2017	√			√			
Chengdu	July 2017	√			√			
Shihezi	Jan. 2017	√	√	√	√			

Sources: Summarized from sub-national government publications of the pioneer cities on LTCI

From the first day of implementation, the performance of these pilot cities has received increasing attention from scholars and policymakers in various countries. Relevant studies have addressed cost issues, including financial efficiency and expenditure forecasting [18, 19]. In addition to the supply-side concerns of balancing budgets, meeting the care needs of individuals and reducing non-essential health service utilization and costs are also key features of successful LTCI. Scholars such as Xueqin Deng and Jin Feng have assessed the policy effects in a pilot city [20, 21], and found that LTCI can effectively reduce the burden on families and healthcare. Some of the studies used publicly available databases to assess the effectiveness of LTCI, but did not accurately correspond to the provisions for the disabled population in each pilot city [22], or did not include the non-covered population in the pilot city in the control group [23]. Therefore, this paper focuses on the effect of LTCI on healthcare utilzation among disabled middle-aged and elderly people, selects appropriate data and methods for accurate analysis and substantive discussion, and explores the heterogeneity of LTCI’s policy effects across regions and different disabled populations from a health equity perspective. On the one hand, it aims to assess the effectiveness of China’s LTCI pilot construction scientifically and quantitatively and provide data support and decision-making reference for the direction and future trend of China’s work to improve LTCI in line with national conditions. On the other hand, it aims to explore the establishment of representative models of LTCI in developing countries and provide a reference for research trends in the field of LTCI.

## Data and methods

### Data sources

The data used in this study were derived from the China Health and Retirement Longitudinal Study (CHARLS). The survey is a large-scale interdisciplinary research project hosted by the National Development Institute of Peking University and jointly implemented by the Survey Centre of the Chinese Academy of Social Sciences of Peking University and the Youth League Committee of Peking University. It uses one-on-one interviews with structured questionnaires to collect a set of high-quality micro-data representing individuals and households of middle-aged people aged 45 and above in China to analyse the ageing of the Chinese population. The questionnaire includes basic personal information, family structure, financial support, health status, physical measurements, health service utilization and health insurance, work, retirement and pensions, income, consumption, assets and basic community information [24]. The CHARLS national baseline survey was conducted in 2011, covering 150 county-level units, 450 village-level units and 17,000 people in approximately 10,000 households. It is followed up every two to three years, with a representative sample and high data quality, and is widely used in Chinese geriatric health research [25]. CHARLS has been approved by the Biomedical Ethics Review Committee of Peking University and assigned the ethical approval number IRB00001052-11015. All participants were required to provide written informed consent.

### Sample selection

**Fig. 1:**
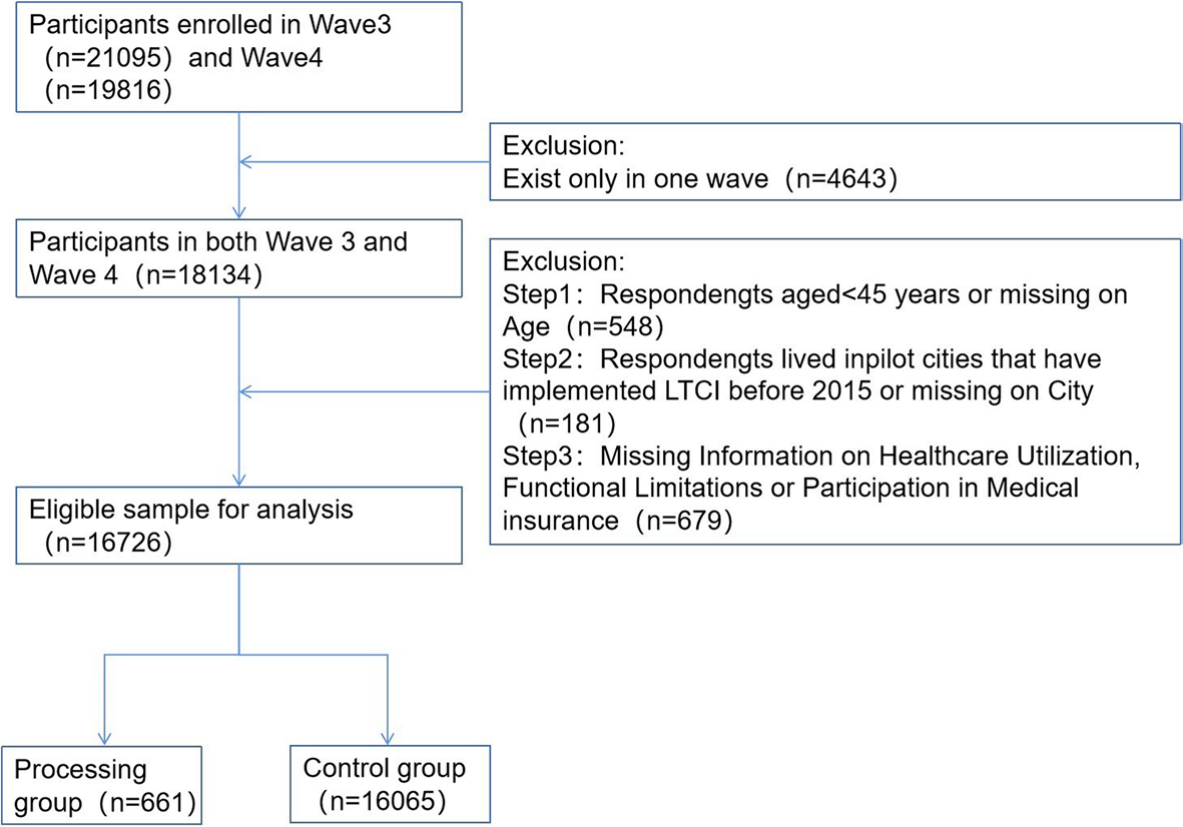
Sample selection process diagram

Panel data from wave 3 (2015) and wave 4 (2018) of CHARLS were selected for analysis in this study, resulting in a two-period strongly balanced panel with 16,726 participants based on the following criteria: 45 years and older and data that provided information on outpatient visits, hospitalizations, participation in medical insurance, and functional limitations in both waves of the study, with the sampling process detailed in Fig. 1. Given that the CHARLS count period is from July to September of the current year, based on matching the cities in which the three-year tracking sample is located to the time when local LTCI policy was implemented, we consider a city to be affected by the policy and in the processing group if local LTCI was implemented more than six months apart from the period of the wave 4 database count (September 2018). Since Qingdao, Weifang and Changchun implemented LTCI in 2012–2015, the samples of the above three cities were deleted from the present study. Combining the policy text and database samples, the present study divides the samples eligible for policy coverage in the pilot cities into three groups based on the type of participation in medical insurance. The first group is the sample of cities that cover only employees’ medical insurance participants, including these participants in Chengde, Qiqihar, Ningbo, Anqing, Shangrao, Jinan, Liaocheng, Binzhou, Guangzhou, Chongqing and Chengdu. The second group is the sample of cities covering employees’ medical insurance participants and urban and rural residents’ medical insurance participants, including these participants in Suzhou, Nantong, Jingmen, and Shihezi, as well as employees’ medical insurance participants and urban residents’ health insurance participants in Jilin. The third group is an age-restricted sample of pilot cities, including all employees’ medical insurance participants in Shanghai, and urban and rural residents’ medical insurance participants over the age of 60. We selected measures of the ability to perform activities of daily living from the CHARLS questionnaire transformed into the Barthel scale, and assessed the degree of disability of individuals based on their scores. The disabled people in the above three groups who were eligible for treatment of LTCI in each location were used as the processing group for the experiment. The sample in the pilot cities that were not covered by the LTCI and the sample of all individuals in the database who were not selected for the pilot cities were used as the control group. A total of 661 participants were assigned to the processing group and 16,065 to the control group. Although the proportion of participants in the processing group was smaller than in the control group, it did not theoretically pose a significant problem for the results: the DID was based on the premise of a common trend and did not require a relative proportion, and the presence of the control group merely provided a counterfactual for the processing group [26].

### Statistical analysis

LTCI is not a one-size-fits-all policy but rather a policy with a pilot nature. The specific timing of the pilot LTCI policy’s introduction is inconsistent across the region, which may be related to the local economic situation, the supply of care services and the degree of ageing. Therefore, implementing LTCI pilots in localities, namely, determining whether or not middle-aged and elderly people are covered by LTCI, is not entirely exogenous and may be a self-selection process. This study aims to address endogeneity and measure accurately the net effect of LTCI on healthcare utilization among the middle-aged and elderly. It chooses a quantitative study based on the difference-in-differences propensity score matching estimator method (PSM-DID), which can be divided into two steps:

- Firstly, PSM was conducted. Based on a set of covariates* x*, the following model was constructed using logit regression:
  1 $${P}_{i} ( x ) = {P}_{r} ( {D}_{i} = 1| {x }_{i}) = Logit[ f ({ x}_{i} ) ],$$
  where *D* is a dummy variable (1 for the processing group and 0 for the control group) and *f(x*_*i*_*)* denotes the linear function of the individual *i* covariate. Propensity scores were calculated by adding to the model, where possible, factors that affect whether an individual participates in LTCI and health service utilization and using logit regression to estimate the propensity score values for each sample. PSM is then performed. The matching results are tested, and the ineligible samples are excluded.
- Secondly, DID was conducted constructing a DID model based on the sample results after PSM and controlling for individual- and time-fixed effects:
  2 $${Y }_{it}= {\beta }_{0} + {\beta }_{1}\ {did}_{it} +{X}_{it} + {\gamma }_{i} +{ \delta }_{t} + {\varepsilon }_{it},$$
  where *i*(*i* = 1, 2, …, n) denotes individuals and *t* (*t* = 2015, 2018) denotes years. *Y*_*it*_ is the dependent variable, indicating the number and costs of outpatient visits in the past month and hospitalizations in the past year for individual *i* in year *t*. *did*_*it*_ is DID estimate and is the interaction term between *t* and *treated* (*did*_*it*_ = t*treated) with *did*_*it*_ = 1 for the processing group after policy implementation and 0 otherwise. *β*_*0*_ denotes the constant term, and *β*_*1*_ is the core coefficient of interest in this study, whereby the effect of LTCI implementation can be examined (i.e. how LTCI affects individual healthcare utilization). *X*_*it*_ is a set of control variables. γ_i_ and δ_t_ are individual- and time-fixed effects, respectively. *ε*_*it*_ is a random error term.

### Variable design

Health service utilization is the dependent variable for this study, and two types of health service utilization collected in the CHARLS database were selected: outpatient visits and hospitalizations. Respondents were asked how many outpatient visits they had made to a health facility in the past month, how much they had spent on them, how many hospitalizations they had received in the past year, and how much they had spent on them. In this study, the number and cost of outpatient visits and the number and cost of hospitalisations were used to assess health services utilization by middle-aged and older people.

The key variable of the study (*did*_*it*_) is a grouped dummy variable, formed by an interaction term (t*treated) consisting of year (*t*) and group (*treated*) information in the questionnaire. The sample with complete participation in the two follow-up surveys and who participated in LTCI between 2015–2018 is set as the processing group, with *did*_*it*_ defined as 1. The rest of the sample was set as the control group, with *did*_*it*_ defined as 0. *t* = 1 indicates the year after policy implementation (2018), and *t* = 0 indicates the year before policy implementation (2015). *treated* = 1 indicates the processing group for the LTCI-covered disabled population sample identified in the previous section in conjunction with the policy text, and *treated* = 0 indicates the control group.

Regarding the control variables, the study relies on the behavioural model of health service utilization proposed by Andersen in 1968 and finally selects three types of variables that influence health service utilization, including predisposing characteristics, enabling resources and health needs [27]. Predisposing characteristics include gender, age, marital status, retirement status, education level and disability status. Enabling resources include urban and rural residence, the geographical distribution of location, personal assets, and available individuals who can help with care. Health needs include self-rated health status, chronic disease status, smoking status and drinking status. The settings and descriptive statistics results of each variable are shown in Table 2. Column (1) shows the statistical characteristics of the full sample, column (2) shows the means of the processing group in 2015, column (3) shows the means of the control group in 2015, and column (4) shows the differences between the means of the processing and control groups and the results of their t-tests in 2015. According to column (4), in 2015 of the baseline survey period, while the differences between the treatment and control groups in the number and cost of outpatient visits and the number and cost of hospitalisations are less significant, there were more significant differences in the means of many control variables. This suggests that there may be some differences in the basic characteristics of the elderly in the processing group and the control group, which might lead to selectivity bias. Therefore, it is necessary to further use PSM to screen out middle-aged and elderly people from the control sample who are similar to the processing group in all aspects, to control for observable differences, and to explore the net effect of LTCI on the utilisation of healthcare services by middle-aged and elderly people.

**Table 2:** Regression model variable settings and descriptive statistics results

Variable	Variable Definitions and Descriptions	(1)	(2)	(3)	(4)
		Full Sample (*n* = 16,726)	Processing Group in 2015 (*n* = 661)	Control Group in 2015 (*n* = 16,065)	t-test for (2)-(3)
		Mean	Mean	Mean	
Key Variable					
Did	Interaction term consisting of a processing group dummy variable and a time dummy variable				
Time	Wave 3 (2015) = 0, wave 4 (2018) = 1				
Treated	Participating LTCI = 1, not participating LTCI = 0				
Dependent Variables					
LMOT	Number of outpatient visits in the past month	0.407	0.463	0.405	1.121
LMOC	Total outpatient expenses in the past month (¥)	258.147	486.110	248.767	2.849***
LYHT	Number of hospitalizations in the past year	0.197	0.216	0.196	0.818
LYHC	Total hospitalization costs in the past year (¥)	1588.443	1916.380	1574.950	0.995
Control Variables					
Gender	Male = 1, Female = 0	0.480	0.495	0.480	0.764
Age	The year of birth of the respondent is used as the starting date and the year of the survey is used as the cut-off date to calculate the age of the respondent as a continuous variable of 45 and above	59.801	60.174	59.785	1.005
Marital	Married = 1, not married = 0	0.873	0.899	0.872	2.036**
Retire	Retired = 1, not retired = 0	0.298	0.340	0.296	2.426**
Education	Illiterate or semi-literate = 1, primary = 2, lower secondary = 3, upper secondary and above = 4	1.923	1.968	1.921	1.162
Live	Classified according to the National Bureau of Statistics of China, rural = 1, urban = 0	0.626	0.283	0.640	-18.802***
Area	West = 1, Central = 2, East = 3	1.962	1.826	1.967	-4.234***
Asset	≤ 700 = 1, 700 ~ 6000 = 2, 6000 ~ 28,713 = 3, > 28,714 = 4	2.454	2.678	2.445	5.823***
Care	Someone helps to look after = 1, none helps to look after = 0	0.701	0.699	0.701	-0.122
Barthel	Self-care = 1, mild functional impairment = 2, Moderate functional impairment = 3, Severe functional impairment = 4	1.227	1.144	1.230	-4.679***
Selfhealth	Very good = 1, good = 2, fair = 3, poor = 4, very poor = 5	2.911	2.825	2.915	-2.372**
Chronic	Number of chronic disease categories, as a continuous variable	1.697	1.694	1.698	-0.055
Smoke	Smoker = 1, nonsmoker = 0	0.285	0.325	0.283	2.343**
Drink	Drinker = 1, nondrinker = 0	0.626	0.750	0.621	6.757***

Note: ^*^P < 0.1, ^**^P < 0.05, ^***^P < 0.01

## Results

### PSM Results

This study first selected a set of covariates that may influence whether an individual is included in LTCI by referring to the Anderson service model and related literature and used 1:2 nearest-neighbour matching within the caliper to match the sample. The basic idea is to find a sample in the control group that matches the covariates of the processing group sample, thus eliminating self-selection bias because of endogeneity and obtaining two samples with approximately the same probability of being included in LTCI. The PSM results were first tested for balance by measuring the standardised mean difference (%bias) of the covariates between the two groups to see whether there was a significant difference in the values of the covariates between the matched individuals. The results of the tests are shown in Table 3. The test results are shown in Table 3. The standardised mean difference of all covariates is less than 10%, and their absolute values are significantly lower than before matching by 17.2%–96.6%. All covariates do not reject the original hypothesis of ‘there is no systematic bias in the values of covariates between the two groups’. The logit regression results before and after matching (Table 4) also showed that the pseudo-R-squared (Ps R2) was significantly smaller after matching and the *p*-value (p > chi2) was no longer significant, indicating that all covariates were better balanced between the processing and control groups after matching, with none of the values taking on much variability and thus not having much influence on the explanatory power of the changes in the explained variables.

**Table 3:** Results of the balance test after PSM

Variable	Matched Mean		%bias	%reduct |bias|	t-test	
	Treated	Control			T	*p* >|t|
Gender	0.4947	0.48941	1.1	65.1	0.27	0.785
Age	61.664	61.43	2.4	38.2	0.62	0.537
Marital	0.89032	0.88918	0.3	96.6	0.09	0.926
Retire	0.36309	0.37557	-2.6	67.7	-0.66	0.506
Education	1.969	2.0079	-3.7	17.2	-0.94	0.345
Live	0.2829	0.25605	5.8	92.5	1.56	0.120
Care	0.72617	0.72504	0.3	92.5	0.07	0.948
Barthel	1.5832	1.5923	-1.6	81.8	-0.43	0.668
_Iarea_2	0.3177	0.30219	3.4	44.6	0.86	0.389
_Iarea_3	0.25416	0.26853	-3.2	83.0	-0.84	0.401
Asset	2.7405	2.7156	2.3	91.2	0.60	0.550
Selfhealth	2.8427	2.8279	1.6	86.7	0.40	0.687
Chronic	1.9425	1.9497	-0.4	78.4	-0.10	0.918
Smoke	0.31467	0.30522	2.1	75.3	0.53	0.599
Drink	0.74357	0.7171	5.7	79.3	1.53	0.125

**Table 4:** Logit regression results before and after matching

Sample	Ps R2	LR chi2	p > chi2	MeanBias	MedBias	B	R	%Var
Unmatched	0.084	933.60	0.000	14.6	8.4	95.5^*^	0.58	17
Matched	0.002	7.48	0.943	2.4	2.3	10.6	0.96	17

Note:^*^ if B > 25%, R outside [0.5; 2]

**Fig. 2:**
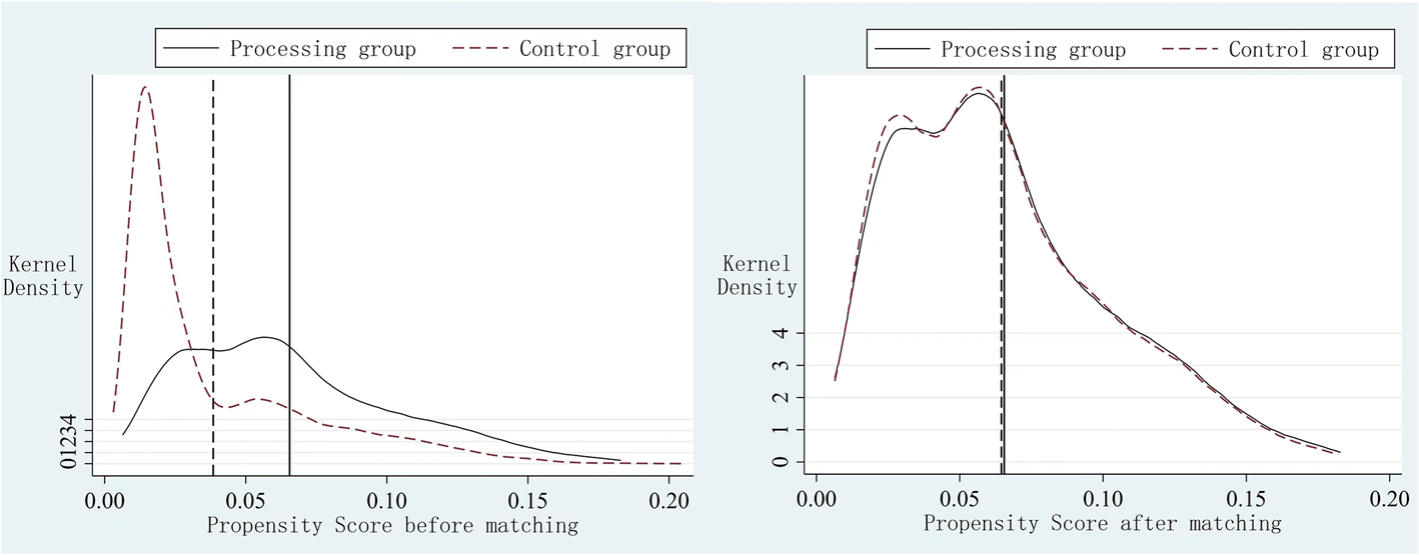
Propensity score values before and after matching

Secondly, a kernel density plot was used to visualise and examine further whether the two groups differed in propensity score values before and after matching (Fig. 2). The kernel density curves of the processing and control groups differed significantly from the mean before matching, whereas they were significantly closer and approximately obeyed a normal distribution after matching. Taken together, the results of the balance tests shown in the above figures and tables indicate that the matching effect is good and that it is more appropriate to use the treated matched sample for DID regression, paving the way for subsequent exploration of the net effect of LTCI on health service utilization by the middle-aged and elderly. Subsequently, to improve the quality of matching of the samples and increase the accuracy and confidence of the estimation results, the areas where the propensity scores of the processing and control groups overlapped were tested, and the samples not undergoing the common support test were excluded, 230 samples in the control group were excluded.

### DID Results

After excluding the samples that were not in the common support test based on the results of PSM, considering that the matched control group samples might act as matches for multiple processing group samples, the control group samples with different weights had different degrees of importance overall. Therefore, we first replicated the samples in the control group that were matched on the basis of the weights and then applied the DID method for frequency-weighted regression to explore the effect of LTCI on the healthcare utilization of middle-aged and elderly people. Finally, 661 samples in the processing group and 5950 samples in the control group participated in the matching and were included in the regression analyses, the regression results are shown in Table 5. The year and group interaction term *did* values for past year hospitalization and costs were − 0.0568101 and − 1236.309, respectively. Both were statistically significant, indicating that the implementation of LTCI was correlated with the annual hospitalization and costs of middle-aged and elderly people in the pilot area and that participation in LTCI reduced the annual hospitalization and costs of middle-aged and elderly people. The interaction term did values for the number of outpatient visits and costs in the past month were − 0.0838251 and − 137.7596, respectively, but were not statistically significant, indicating that LTCI did not significantly change the number of monthly outpatient visits and costs of middle-aged and elderly people in the pilot area.

**Table 5:** Frequency-weighted regression DID results

Variables	LMOT	LMOC	LYHT	LYHC
Did	-0.0838251 (0.0570385)	-137.7596 (150.1579)	-0.0568101^**^ (0.0277182)	-1236.309^**^ (502.0659)
Controlling variables	√	√	√	√
Individual fixed effects	√	√	√	√
Time fixed effects	√	√	√	√
Sample size	6611	6611	6611	6611
R-squared	0.8905	0.6992	0.8776	0.9186

Note: ^*^P < 0.1, ^**^P < 0.05,^***^ *P* < 0.01, Standard errors are in brackets

### Robustness test results

This study also conducted robustness tests to verify the reliability of the above results further:

1. Consideration of data outliers. Given the small sample size of the processing group in this study, the chance behaviour of a single individual is likely to introduce large fluctuations in the mean level. To eliminate the effect of extreme values on the results, the maximum values of the continuous variables in the dependent variable were all winsorised at the top 1% level, and the DID regressions in Table 5 were repeated, as shown in the top half of Table 6, with robust results. Moreover, given the more extreme values of past month outpatient costs and past year hospitalization costs, taking the logarithm also reduces sensitivity to their outliers, as shown in the bottom half of Table 6, where the results remain robust when adding 1 to the logarithm of outpatient costs in the past month and hospitalization costs in the past year. The negative effect of LTCI on the number and costs of annual hospitalizations is statistically significant, and the results of the robustness tests obtained by eliminating the effect of extreme values still support the above findings. Meanwhile, LTCI has a non-significant and robust effect on the number and costs of monthly outpatient visits. The later section will focus on its effect on annual inpatient medical services.
2. Conducted a placebo test. Considering that changes in health care costs may be because of external shocks rather than the implementation of LTCI, this study also used a placebo test to dispel any doubts about the effect of other regional policies. The same DID regressions were conducted using health insurance claims information for outpatient visits in the past month and hospitalizations in the past year as the dependent variables. Columns (1) and (3) of Table 7 show that hospitalization reimbursement expenses decreased and were significantly correlated with participation in LTCI and that outpatient reimbursement expenses showed some increase but no statistically significant correlation with LTCI. Although columns (2) and (4) show that the reimbursement rates for hospital hospitalization and outpatient expenditures remained almost unchanged, no significant difference is observed in health insurance policies on hospitalization and outpatient services in the processing group compared with the control group. LTCI in the previous conclusion effectively reduced expenditure on hospitalization for middle-aged and elderly people not because of changes in other coverage entitlements in the processing group compared with the control group but simply because of the presence of LTCI.

**Table 6:** DID results after scaling and taking logarithms

Variables	LMOT	LMOC	LYHT	LYHC
Winsorising 1% of maximum	-0.0414833	63.98176	-0.0545342^**^	-1274.411^***^
	(0.0371681)	(35.521)	(0.0219211)	(302.175)
Taking the logarithm of the dependent variable	-	-0.0409492	-	-0.463204^***^
	-	(0.1070045)	-	(0.1454059)
Controlling variables	√	√	√	√
Individual fixed effects	√	√	√	√
Time fixed effects	√	√	√	√
Sample size	6611	6611	6611	6611

Note: ^*^P < 0.1,^**^ *P* < 0.05, ^***^ *P* < 0.01, Standard errors are in brackets

**Table 7:** Results of DID regression analysis of the effect of LTCI on health insurance reimbursement costs and reimbursement rates

Variables	(1)	(2)	(3)	(4)
	Outpatient medical insurance reimbursement	Outpatient medical insurance reimbursement rates	Hospitalization medical insurance reimbursement	Hospitalization medical insurance reimbursement rates
Did	84.31406 (54.67146)	0.0118468 (0.0369012)	-909.4944^***^ (248.7694)	0.0051313 (0.03292)
Controlling variables	√	√	√	√
Individual fixed effects	√	√	√	√
Time fixed effects	√	√	√	√
Sample size	6,611	1,233	6,611	1,085
R-squared	0.7605	0.9699	0.9551	0.9831

Note: ^*^P < 0.1, ^**^P < 0.05, ^***^P < 0.01, Standard errors are in brackets

### Heterogeneity test results

The effect of LTCI on healthcare utilization may be heterogeneous for different groups with different characteristics, and heterogeneity analysis can further explore the effect of LTCI on healthcare utilization among different groups of middle-aged and elderly people, as well as understanding the status of LTCI use among beneficiaries, which can be used to make relevant policy recommendations. Considering the characteristics of the sample, this study examines sub-groups of middle-aged and older people in terms of geographical distribution and educational level and care assistance, respectively. The regression results are reported in Table 8. The coefficients on the number and costs of visits in the past month are insignificant across subgroups and, therefore, not reported.

**Table 8:** Results of the heterogeneity test of LTCI on healthcare expenditures

Variables	LYHT				LYHC			
	Eastern	Central and Western	Low education level and unattended	Other	Eastern	Central and Western	Low education level and unattended	Other
Did	-0.104^**^ (0.048)	-0.046 (0.053)	0.155 (0.095)	-0.047^*^ (0.028)	-3359.863^***^ (1242.883)	301.189 (710.387)	332.611 (423.594)	-653.418^*^ (342.384)
Controlling variables	√	√	√	√	√	√	√	√
Individual fixed effects	√	√	√	√	√	√	√	√
Time fixed effects	√	√	√	√	√	√	√	√
Sample size	2562	4189	1190	5292	2562	4189	1190	5292
R-squared	0.888	0.822	0.966	0.920	0.838	0.843	0.994	0.890

Note: ^*^P < 0.1,^**^ *P* < 0.05, ^***^ *P* < 0.01, Standard errors are in brackets

The effect of the region was first examined by dividing the original sample into two sub-samples, central and western regions and eastern region, for regression. The results showed that both sub-samples had a negative effect on the number of annual hospitalizations. However, this effect was significant for the eastern region population and insignificant for the central and western regions population. Regarding annual hospitalization costs, LTCI had a negative and statistically significant effect in the eastern region and a positive but non-significant correlation in the central and western regions. The original sample was then regrouped into two sub-samples, low education level and unattended and other conditions, to examine the effect of individual education and care assistance. The results showed that LTCI had no significant effect on low education and unattended middle-aged and older people but reduced the number and cost of annual hospitalizations for other middle-aged and older people.

## Discussion and suggestion

### Effect of LTCI on health service utilization of middle-aged and elderly people

Similar to previous studies [28, 29], the results of this study also suggest that LTCI effectively reduces hospitalization health service utilization among middle-aged and elderly adults. Unlike the study by Chao Ma et al., this study found that the effect of LTCI on outpatient health service utilization was not significant, possibly because of the difference in the chosen study sites. Chao Ma et al. studied the effectiveness of policy implementation of LTCI in Qingdao, which was the first the LTCI pilot city to explore implementation programmes in 2012, three to five years earlier than other cities. Over the past few years, a series of complementary policies have been introduced to develop care services that cover a wide range of population groups in various forms, providing timely, continuous and integrated long-term care services for insured persons. Some policies also direct the people at risk of mild disability to preventive care, improves the health of insured persons and makes them less in need of outpatient medical services [30]. In addition, considering that during the same period China was implementing a reform to integrate the NCMS and the URBMI into the urban–rural health insurance. We conducted a relevant literature search, and most of the studies exploring the impact of urban–rural health insurance integration showed that the policy increased the utilization of healthcare services for rural residents and had a less significant impact for urban residents [31, 32]. Combined with the results of the analyses in this study, it somewhat reinforces the idea that LTCI is effective in reducing inpatient healthcare service utilization among middle-aged and older adults.

Analysing the regression results obtained in this study, the possible reason is that the care system provided by LTCI and the corresponding entitlement payments have effectively guided the disabled middle-aged and elderly people to accept the long-term care service programme, thus reducing some of the original demand for hospitalisation. The pilot areas will provide home care services and enable disabled patients to stay in professional or elderly care institutions with medical qualifications to build a ‘medical, nursing, care and recreation’ system that relies on families and communities. The medical expenses incurred will be reimbursed proportionally, while the fees for nursing services will be settled by social security institutions and nursing care institutions on a fixed lump-sum basis [33], leading to the direct transfer of many disabled elderly people who have been hospitalised. Some pilot cities have also specified that participants are not entitled to medical insurance treatment for hospitalization while receiving reimbursement for LTCI to reduce the excessive utilization of medical resources and encourage some patients who need care or rehabilitation through hospitalization to turn to home care [34, 35], thus effectively alleviating the problem of ‘social hospitalization’ and reducing medical costs. The non-significant effect of LTCI on outpatient service utilization may be because of the following reasons. On the one hand, the relatively low average cost of outpatient care imposes a low financial burden on patients; when faced with the need for outpatient services, patients and their families may trust the services the hospital provides more than a new system of care because of its authority. On the other hand, implementing LTCI focuses on addressing the long-term care needs of the severely disabled. Most of the pilot areas are compensated for disabled people who need long-term care after more than six months of treatment [36], and they have less demand for outpatient care and more demand for daily living care. Thus, the utilization of outpatient services for the middle-aged and elderly has little difference before and after the implementation of LTCI.

Based on the previous description of the population covered in the pilot cities and the results of the statistical analyses, this study makes some recommendations. Firstly, a scientific rating standard and assessment system for a disability should be developed, and a reasonable catalogue and reimbursement contents should be designed to meet the local economic level. The policy documents of the first pilot cities of LTCI in China did not explicitly provide for a unified disability rating standard, and the pilot cities mainly adopted the Barthel Scale and the locally-developed comprehensive scale as the tools for assessing the degree of incapacity. To emphasise the differences in local LTCI, the pilot cities tend to ignore the universal rules of the social insurance system, and the path dependency resulting from long-term trial and error will increase the cost of policy unification. Therefore, the results of each pilot project should be evaluated immediately, summarizing universal standards and experiences to provide referable principle-based standards for the further development of more pilot LTCI programmes in China. At the same time, each pilot region should reasonably choose a long-term care service model according to the different local economic and characteristics of the disabled, improve service facilities and service conditions and increase the reserve of care resources, so that a nationwide LTCI system can eventually be established. Secondly, LTCI should be integrated into the multi-layered, multi-pillar social security system. Life care and primary medical care should be combined, and the provision that participants can enjoy LTCI treatment and outpatient coordination treatment paid by the medical insurance fund should be improved, so as to adapt to the context of the establishment of a multi-level medical insurance system. Resources for long-term care services should be increased to alleviate the delayed discharge of disabled persons, pressurised patients and medical care instead of nursing to improve the health of people with disabilities and reduce their outpatient and inpatient care burden. Finally, the pilot coverage should be further expanded in due course, and the conditions for LTCIs should be gradually relaxed. At present, LTCI in the pilot areas mainly covers people with severe disabilities who participate in urban employees’ medical insurance and can be appropriately expanded to cover all people with mild, moderate and severe disability and people with dementia among urban employees’ medical insurance and urban and rural medical insurance participants. The successful pilot experience of LTCI in the first batch of pilot cities should also be extended to more cities as soon as possible to alleviate the worsening level of disability among the middle-aged and elderly to meet the care needs arising from an ageing population and save LTCI funds and medical insurance fund expenditures effectively.

### Regional, educational level and assistance in care heterogeneity in the effect of LTCI on health care utilization among middle-aged and older adults

The results of the heterogeneity analysis show that the effect of LTCI on the utilization of residential services for middle-aged and elderly people in the eastern pilot areas is more pronounced in terms of geographical distribution, which differs from the results of Yanzhe Zhang and Xiao Yu’s study in which residents in central and western inland cities were more accepting of LTCI than those in eastern coastal cities [37], probably because the subjective satisfaction with LTCI is higher in the central and western regions with a high net outflow of population and a higher proportion of elderly people living alone with empty nesters have more urgent care needs.

LTCI’s significant impact on the utilization of hospitalizations for middle-aged and elderly adults in the eastern region is analysed as follows. Firstly, given that China has entered an ageing society, regional inequalities in population ageing have become increasingly prominent in recent years [38], with the eastern region having a more serious ageing population and a relatively high level of economic and social development, where residents are well-educated in insurance culture and choose health insurance to transfer risks when encountering health risks and are therefore more receptive to the new concept of LTCI and the combination of medical and health care [39]. Secondly, the economic level of the central and western regions is lower, people’s purchasing power is weaker, and the pressure on the medical insurance fund is greater, whereas the eastern region is economically developed and have high financial levels. Therefore, the coverage and services provided by LTCI policies in the central and western regions and the eastern regions have a large gap [40], the eastern regions offer a wider range of coverage, more professional medical, more nursing and elderly care institutions and residents’ higher ability to pay. Thus, middle-aged and older people in the east benefit more from LTCI.

The heterogeneity analysis also found that LTCI has a non-significant effect on hospitalizations utilization among low-education and unattended middle-aged and older people but reduced the number and cost of annual hospitalizations among other middle-aged and older adults. Analysing the reasons, most middle-aged and elderly people are influenced by the traditional Chinese filial culture and choose home-based care for the aged, relying heavily on informal care from family members [41], whereas LTCI treatment is mainly based on reimbursement for institutional care and home care services provided by them. Middle-aged and elderly people with a high level of education and their caregivers may choose formal care services to reduce the double financial burden of the cost of elderly care and their family’s financial situation and obtain more working hours [42], thus reducing hospitalizations. The lower-educated and unattended middle-aged and elderly adults, usually in poorer financial and health conditions, still have more health needs after joining LTCI, have more difficulty distinguishing between care needs and medical needs, and tend to choose hospitalization for treatment when they have health needs.

Based on the above findings, the following recommendations are made in this study. Firstly, the LTCI system should be improved according to local conditions on the basis of summing up the pilot experiences of each region. Influenced by the scope of the pilot, the level of financial support and the balance between supply and demand, the effects of LTCI have regional differences, being more pronounced in the eastern region compared to the central and western regions. Policies should be formulated and implemented to avoid the inequitable provision of protection and care services with a focus on the central and western regions. Policies should improve the intensity of LTCI compensation and the quality of care services, combine transfer payment methods to ensure fairness and equity between different regions and make differentiated designs in terms of the characterization of the system, funding methods and treatment methods, considering the level of regional economic development. Secondly, the referral interface between medical and skilled nursing facilities must be improved and the capacity of skilled nursing facilities must be strengthened to promote LTCI and health literacy content. Influenced by the culture of filial piety, many middle-aged and elderly people choose home-based care for the aged. Some of them are not always accompanied by someone to care for them, and their education level is not high enough to understand the knowledge and usage of new things like LTCI, so they still prefer hospitals for treatment when they encounter health problems. Thus, a referral mechanism must be established between medical institutions and skilled nursing facilities for those involved in LTCI disabilities, and policy advocacy and health literacy among family caregivers and skilled nursing staff in medical care facilities should be enhanced for this group of middle-aged and elderly adults.

### Limitation

This study has two limitations. First, the study period covered only two waves due to the limitations of the publicly available database. The short study period may not reflect the long-term impact of long-term care insurance on health care use. The follow-up research team will continue the study after the release of the new wave of survey data. Second, given the limitations of the sample size, further validation is needed. The research team will subsequently build on this study to find more partners to obtain data on LTCI coverage and use cost–benefit analyses to provide a clearer picture of the overall benefits.

## Conclusions

This study has two main contributions. Firstly, after exploring the effect of LTCI on the utilization of health care services for middle-aged and elderly adults in the pilot cities, LTCI is found to reduce the utilization of hospitalisations but did not significantly affect the utilization of outpatient services. Moreover, policymakers need to improve the treatment plans of LTCI further and increase the supply of resources for long-term care services. Secondly, a heterogeneity analysis revealed that the effect of LTCI on the utilization of hospitalizations by middle-aged and elderly adults in the pilot cities differed in terms of regional distribution and educational level and care assistance with health service utilization by middle-aged and elderly adults in the central and western regions and those with low educational levels or unattended care insignificantly affected by LTCI. Policymakers should develop locally tailored long-term care service models to facilitate the use of long-term care resources by different disabled middle-aged and elderly people in different regions, based on the actual situation of the economy, medical resources and health service needs of middle-aged and elderly adults in different regions. Our findings are informative for the literature on ageing societies, social sector reform and policy evaluation. Moreover, China’s experience could provide useful lessons for other developing countries considering similar LTCI to strengthen their response to population ageing.

## Data Availability

The datasets generated and analyzed during the current study are available in the CHARLS repository, [http://charls.pku.edu.cn/en].

## Abbreviations

LTCI: Long-term care insurance
PSM-DID: Difference-in-Differences Propensity Score Matching estimator
CHARLS: China health and retirement longitudinal study
PSM: Propensity Score Matching
DID: Difference-in-Differences estimator
